# Feasibility of Randomized Controlled Trials for Cancer Drugs Approved by the Food and Drug Administration Based on Single-Arm Studies

**DOI:** 10.1093/jncics/pkab061

**Published:** 2021-06-30

**Authors:** Rebekah Rittberg, Piotr Czaykowski, Saroj Niraula

**Affiliations:** 1 Section of Hematology/Oncology, Department of Internal Medicine, University of Manitoba, Winnipeg, MB, Canada; 2 Department of Medical Oncology and Hematology, CancerCare Manitoba, Winnipeg, MB, Canada; 3 Department of Community Health Sciences, University of Manitoba, Winnipeg, MB, Canada

## Abstract

**Background:**

The US Food and Drug Administration (FDA) introduced an Accelerated Approval (AA) pathway to expedite patient access to new drugs. AA accepts less rigorous trial designs, including single-arm studies (SAS), owing to perceived lack of feasibility of timely randomized controlled trials (RCTs).

**Methods:**

We designed hypothetical RCTs with endpoints of overall response rate (ORR), progression-free survival (PFS), and overall survival (OS) for FDA approvals based on SAS for solid tumors during 2010-2019. Existing standards of care served as controls. RCTs were designed to detect a difference with power of 0.80, α-error of 5% (2-sided), and 1:1 randomization. Accrual duration was estimated based on participation by less than 5% of eligible patients derived from cancer-specific incidence and mortality rates in the United States.

**Results:**

Of 172 (18.0%) approvals during the study period, 31 (18.0%) were based on SAS. Median sample size was 104 (range = 23-411), and 77.4% were AA. All studies reported ORR, 55% reported duration of response, 19.4% reported PFS, and 22.5% reported OS. Median sample sizes needed to conduct RCTs with endpoints of ORR, PFS, and OS were 206, 130, and 396, respectively. It would have been theoretically possible to conduct RCTs within duration comparable with that required by SAS for 84.6%, 94.1%, and 80.0% of approvals with endpoints of ORR, PFS, and OS, respectively.

**Conclusion:**

An overwhelming majority of FDA approvals based on SAS should be feasible as RCTs within a reasonable time frame. Given the collateral harms to patients and to scientific rigor, drug approval based on SAS should only be permitted under exceptional circumstances.

To ensure that new drugs entering the market are safe and effective, the US Food and Drug Administration (FDA), the federal agency for drug regulation, has historically relied on results from randomized controlled trials (RCTs), the gold standard method to evaluate efficacy and toxicity of new health interventions compared with existing standards of care. However, conventional drug development with 3 sequential phases of clinical trials is resource intensive, typically requiring hundreds of millions of dollars and more than a decade to complete (1,2). This can substantially delay the time for a promising new drug to move from the bench to the bedside.

As a way to expedite access to new drugs for patients, the FDA introduced an Accelerated Approval (AA) pathway for drug approval in 1992 for serious or life-threatening diseases (3). This pathway allows for “conditional” approval of promising new drugs based on less rigorous evidence than that required for full approvals so that patients have early access to these drugs while definitive evidence is being generated (4).

In the last few years, there has been an increase in drugs receiving accelerated FDA approvals based on single-arm studies (SAS) (5). Although FDA guidance states explicitly that SAS are acceptable for AA “in settings where there is no available therapy and where major tumor regressions can be presumed to be attributed to the tested drug” (https://www.fda.gov/media/71195/download), SAS have been used frequently despite the existence of reasonable alternate therapy. Notably, “major tumor regression” can hardly apply for examples such as avelumab in urothelial carcinoma where approval was based on an overall response rate (ORR) of a mere 13% (lasting 2 months, which is typically the duration to first radiological reevaluation). Indeed, duration of response is not specified in FDA guidance as a basis to use SAS for approvals.

SAS do not possess the methodological rigor of RCTs but are useful typically in initial phases of drug development mainly to establish the dose and to evaluate preliminary antitumor activity and safety. Resulting from the absence of a control arm, SAS cannot directly measure if a drug improves outcomes compared with existing standards of care. Despite these drawbacks, SAS are accepted increasingly as proof of efficacy of drugs in those circumstances when standard treatments either do not exist or are clearly inferior to the new treatments, when the disease is rare, and in any situations where patient accrual for RCTs is not perceived to be feasible (6). Although use of SAS in these circumstances is reasonable, the use of SAS is not always based on empiric evidence of infeasibility of RCTs, hence, inviting opportunity for questionable application of SAS perhaps driven by associated economic incentives to a manufacturer.

Here, we review the last 10 years of FDA approvals for solid tumors based on SAS and assess the feasibility of conducting RCTs for those approvals using the same endpoints used by the corresponding SAS.

## Methods

### Data Source and Search Strategy

The US FDA website was accessed for review of oncology drugs approved for solid tumors from January 1, 2010, until December 31, 2019. Pivotal trials used to support each approval were identified from the FDA drug labels and corresponding primary publications assessed. Only the drugs approved on the basis of SAS were included. Two authors collected the data independently (RR, SN), and any discrepancies were resolved by consensus. From the FDA website and from the primary publications, we collected the following information for each drug: date of approval, indication, line of therapy, approval pathway (AA or regular), number of patients accrued, and the outcomes reported (overall response rate [ORR], duration of response [DOR], progression-free survival [PFS], overall survival [OS]).

We then assessed the clinicaltrials.gov website to extract information on SAS start date and completion date. Duration of time required to conduct each SAS was then determined using these dates and verified using information in FDA notification, first journal publication, and/or conference presentation.

Lastly, we used the Surveillance, Epidemiology and End Results (SEER) website to obtain malignancy-specific information and annual number of deaths for the cancer type (or subtype) involved. Potential number of patients with these conditions who would be eligible for clinical trials was approximated conservatively as 5% of the number of patients who dies from the given condition annually in the United States (evidence suggests that 5% to 16% of eligible patients enroll into clinical trials in the United States) (7). When appropriate, prevalence of disease-specific mutations was accounted for in the cancer subgroup.

### Statistical Analysis

Based on the above information, we then designed (hypothetical) RCTs using PS: power and sample size calculation software version 3.6.3 (Vanderbilt University, Nashville, TN) (8). All RCTs were designed using conventional assumptions used by contemporary trial designs including power (1-ß, where ß is type II error) of 0.80, α-error (type I error) of 5% (2-sided), and 1:1 randomization (9). All outcomes (ORR, PFS, and OS) reported in the SAS were used as the primary endpoints of hypothetical RCTs separately to assess feasibility of RCTs with corresponding primary endpoints. Outcomes reported in the literature for existing standard of care, for the same condition, were used for the control group. Where more than 1 standard of care existed, we used the treatment associated with the best outcome. Where no apparent standard of care existed for a condition, best estimate of the outcome (eg, survival) for control group was obtained by polling at least 2 board-certified experts specializing in the disease condition. Follow-up duration for the time-to-event endpoints (PFS and OS) for the hypothetical RCTs was set conservatively as twice the median life expectancy for the disease condition. When PFS was not reported, we used DOR as a surrogate for PFS when available. To avoid overly optimistic assessment for feasibility of RCTs, the most conservative assumptions were used, such as use of low percentage of eligible patients for enrollment in RCTs and twice the life expectancy as the expected median survival duration.

## Results

### Search Results and Approval Characteristics

**Figure 1.**
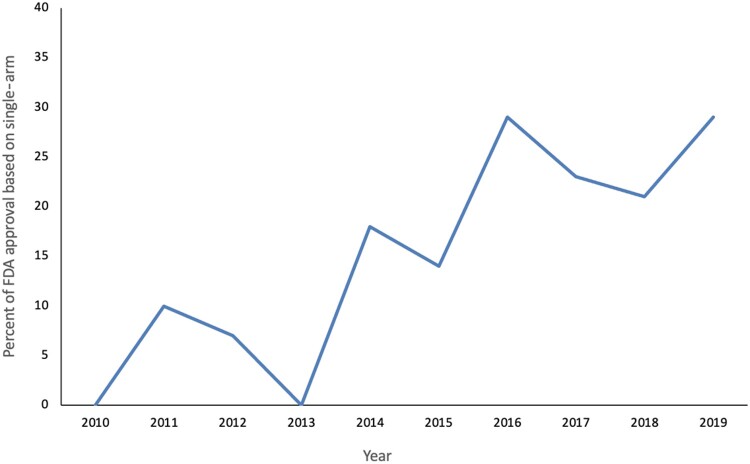
Percentage of US Food and Drug Administration (FDA) approvals based on single-arm data between 2010 and 2019.

Between 2010 and 2019, the FDA approved 172 unique anticancer drug indications of which 31 (18.0%) were for solid tumors based on SAS (Table 1). The absolute number of SAS increased from 0 in 2010 to 8 in 2019 (Figure 1). All approvals were for metastatic settings and based on ORR as primary endpoint. The pathway for drug approval was AA for 77.4% (24 of 31) of drugs and full approval for the remainder. ORR was reported in 100% of SAS, DOR in 54.8%, PFS in 19.3%, and OS in 22.6% of the included SAS, after review of all publications pertaining to the SAS. Median sample size of involved SAS was 104 (range = 23-411) patients per approval. Drugs were approved based on a median ORR of 39% (range = 13%-78%). ORR for existing standard of care was 23% (range = 5%-62%).

**Table 1.** Single-arm studies approved by Food and Drug Administration between 2010 and 2019 with estimated RCT sample size for ORR, PFS, and OS endpoints^a^

Year	Drug	Indication	Standard of care	Line of therapy	SAS sample size	ORR, %	AA	Progression free survival/Duration of response, mo	Duration of SAS, y	No. of patients eligible per year	Estimated RCT sample size with ORR endpoint (both arms)	Estimated RCT sample size with PFS endpoint (both arms)	Estimated RCT sample size with OS endpoint (both arms)
2019	Trastuzumab Deruxtecan	Metastatic breast cancer, HER2 positive	Neratinib and capecitabine	3rd or later	184	60.3	Yes	14.8	2.3	6326	114	78	—
2019	Enfortumab vedotin-ejfv	Metastatic urothelial cancer	Cytotoxic chemotherapy (CT)	2nd or later	125	44	Yes	7.6	2.2	17 980	46	46	—
2019	Niraparib	Ovarian, fallopian tube or primary peritoneal cancer, with homologous recombination deficiency	Cytotoxic CT	3rd or later	98	24	No	8.3	3.2	2091	—	204	—
2019	Pembrolizumab plus lenvatinib	Advanced endometrial cancer, not MSI-H or dMMR	Cytotoxic CT	2nd or later	108	38.3	Yes	Not reached	3.7	9443	572	—	—
2019	Entrectinib	Metastatic NTRK solid tumors	Variable	2nd or later	54	57	Yes	10.0	3.7	1880	148	—	—
2019	Entrectinib	Metastatic NSCLC, ROS-1 positive	Cytotoxic CT	2nd or later	51	78	Yes	10.4	3.7	1154	44	56	—
2019	Pembrolizumab	Metastatic SCLC	Cytotoxic CT	3rd or later	83	19	Yes	Not reached	5.3	13 572	194	—	—
2019	Erdafitinib	Metastatic urothelial cancer, susceptible FGFR3 or FGFR2 genetic alterations	Cytotoxic CT	2nd or later	87	32.2	Yes	5.4	3.9	13 596	276	130	—
2018	Pembrolizumab	Advanced Merkel cell carcinoma	Cytotoxic CT	1st	50	56	Yes	16.8	3.2	708	—	10	—
2018	Larotrectinib	Metastatic NTRK solid tumors	Variable	2nd or later	55	75	Yes	Not reached	4.6	1880	52	—	—
2018	Pembrolizumab	Hepatocellular carcinoma	Cytotoxic CT	2nd or later	104	17	Yes	4.9	2.5	12 667	1114	150	954
2018	Iobenguane I 131	Advanced pheochromocytoma or paraganglioma	Cytotoxic CT	1st or later	68	22	No	Not reached	5.1	818	—	—	—
2018	Pembrolizumab	Advanced cervical cancer, high PD-L1	Cytotoxic CT	2nd or later	98	14.3	No	Not reported	2.5	1502	—	—	—
2018	Dabrafenib plus trametinib	Metastatic anaplastic thyroid cancer with BRAF V600E mutation	Cytotoxic CT	1st or later	23	61	No	Not reached	4.1	338	674	—	—
2017	Nivolumab	Metastatic colorectal cancer with MSI-H or dMMR	Cytotoxic CT	2nd or later	74	32	Yes	Not reached	3.4	6384	450	—	—
2017	Pembrolizumab	Metastatic colorectal cancer with MSI-H or dMMR	Cytotoxic CT	2nd or later	149	39.6	Yes	Not reached	1.5	6384	1724	—	—
2017	Avelumab	Metastatic urothelial carcinoma	Cytotoxic CT	2nd or later	242	13.3	Yes	2.7	4.3	17 980	422	—	—
2017	Durvalumab	Metastatic urothelial carcinoma	Cytotoxic CT	2nd or later	191	17	Yes	Not reached	4.7	17 980	216	—	114
2017	Avelumab	Metastatic Merkel cell carcinoma	Cytotoxic C	2nd or later	88	32	Yes	Not reached	2.8	708	1386	348	392
2017	Nivolumab	Metastatic urothelial carcinoma	Cytotoxic CT	2nd or later	270	19.6	Yes	10.3	1.9	17 980	182	24	738
2016	Rucaparib	Ovarian cancer, BRCA positive	Cytotoxic CT	3rd or later	106	54	Yes	9.2	5.1	2091	120	128	—
2016	Pembrolizumab	Metastatic head and neck SCC	Cytotoxic CT	2nd or later	174	16	Yes	6.9	1.5	10 750	1050	160	—
2016	Atezolizumab	Urothelial cancer	Cytotoxic CT	2nd or later	310	14.8	Yes	Not reached	1.0	17 980	330	—	—
2016	Crizotinib	Metastatic NSCLC, ROS-1 positive	Cytotoxic CT	1st or later	50	66	No	18.3	6.4	1154	82	140	20
2015	Alectinib	Metastatic NSCLC, ALK rearrangement	Cytotoxic CT	2nd or later	225	44	Yes	11.2	1.1	6785	166	48	—
2015	Osimertinib	Metastatic NSCLC, EGFR mutation (T790M)	Cytotoxic CT	2nd or later	411	59	Yes	12.4	1.5	16 965	64	338	—
2015	Pembrolizumab	Metastatic NSCLC, high PD-L1	Cytotoxic CT	2nd or later	61	41	Yes	Not reached	1.4	65 146	218	—	—
2014	Olaparib	Ovarian cancer, BRCA positive	Cytotoxic CT	4th or later	137	34	No	7.9	2.4	2091	848	712	—
2014	Ceritinib	Metastatic NSCLC, ALK rearrangement	Cytotoxic CT	2nd or later	163	44	Yes	7.1	1.3	6785	64	228	—
2012	Vismodegib	Locally advanced/metastatic basal cell carcinoma	Best supportive therapy	1st or later	96	30.% (mBCC) 42.9 (laBCC)	No	7.6 for both	1.7	1000	—	—	—
2011	Crizotinib	Metastatic NSCLC, ALK rearrangement	Cytotoxic CT	2nd or later	136	61	Yes	11.0	2.0	6785	34	50	—

a AA = accelerated approval; BRCA = breast cancer gene; CT = computerized tomography; dMMR = deficient mismatch repair; FGFR = fibroblast growth factor receptor; laBCC = locally advanced basal cell carcinoma; mBCC = metastatic basal cell carcinoma; MSI-H = microsatellite instability-high; NSCLC = non-small cell lung cancer; NTRK = neutropenic tyrosine receptor kinase; OS = overall survival; ORR = overall response rate; PD-L1 = programmed death ligand 1; PFS = progression-free survival; RCT = randomized controlled trials; ROS-1 = ROS proto-oncogene 1; SAS = single-arm studies; SCC = squamous cell carcinoma; SCLC = small cell lung cancer.

Out of 31 drugs, 5 (16.1%) were tested as first-line therapy and 26 (83.9%) of 31 as second-line therapy or later. Prevalence of cancer subtype had no correlation with frequency of approvals using SAS: non-small cell lung cancer (NSCLC) accounted for the most frequent (22.6%) of SAS drug approvals, followed by urothelial carcinoma (19.3%) and ovarian cancer (9.6%). Immunotherapy accounted for 45.2% (14 of 31) and kinase inhibitors 35.5% (11 of 31) of approvals. Pembrolizumab was the single most common drug evaluated in 25.8% (8 of 31) of approvals. Only 2 (6.5%) of the 31 studies evaluated a combination treatment, with the remainder evaluating single agents.

Drugs for 4 (12.9%) indications were approved despite a lower ORR compared with existing standard of care, and 1 (3.2%) drug was approved with shorter DOR compared with existing standard of care. Furthermore, these approvals also lacked any apparent alternate advantage compared with existing standard of care such as improvement in quality of life, cost, or convenience of treatment. Overall, 7 (22.6%) approvals based on ORR were regular approvals, requiring no post-marketing clinical trials.

### RCTs With ORR as Primary Endpoint

All SAS reported ORR, with 87.1% (27 of 31) demonstrating a higher ORR compared with previous standard of care, allowing us to design hypothetical RCTs for these approvals with ORR as the primary endpoint. ORR for vismodegib for advanced or metastatic basal cell carcinoma was excluded because of a lack of an appropriate control arm (ie, previous standard of care). The median sample size needed to conduct RCTs with ORR as the primary endpoint, when using the control arm ORR from the previous standard of care, was 206 (range = 34-1724) for both arms combined. The sample size for 30.8% (8 of 26) of the SAS was larger than what would be necessary to complete an RCT. Based on a conservative accrual rate of 5% of the potentially eligible population, 57.7% (15 of 26) of the hypothetical RCTs could have been completed within an accrual period of 12 months and 84.6% (22 of 26) within a 24-month period. Of such RCTs, 81% (21 of 26) would have shorter accrual times than the duration that was required to complete the corresponding SAS.

### RCTs With PFS as Primary Endpoint

Absolute PFS duration could be extracted for 61.3% (19 of 31) of approvals, after review of all abstracts and full publications pertaining to the SAS. We excluded 2 approvals from designing RCTs with PFS endpoints: vismodegib for advanced or metastatic basal cell carcinoma (lack of control arm) and entrectinib for metastatic neutropenic tyrosine receptor kinase solid tumors (PFS in the reported SAS was notably shorter than that reported with the existing standard of care) (10). For the remaining 17 SAS with PFS information, the median sample size needed to detect a statistically significant difference in PFS in RCTs was 130 (range = 10-712) for both arms combined. Based on an accrual rate of 5% of the eligible population, 94.1% (16 of 17) of the RCTs could have been completed within the time frame required for the corresponding SAS, and 88.2% (15 of 17) could have been completed within 24 months.

### RCTs With OS as Primary Endpoint

Duration of OS was reported for 22.5% (7 of 31) of approvals, of which appropriate control arms for metastatic neutropenic tyrosine receptor kinase solid tumors and advanced pheochromocytoma and/or paraganglioma could not be reliably estimated. For the remaining 5 SAS that provided OS information, the median sample size needed to detect a statistically significant difference with OS as primary endpoint was 392 (range = 20-954) for both arms. Accrual for 4 of 5 (80.0%) SAS could have been completed within the time frame needed to complete the SAS; all 5 could have been completed within 24 months (Table 1).

## Discussion

Lack of feasibility for timely completion of RCTs is the primary assumption supporting the rationale for FDA AA based on less rigorous clinical trials like SAS. In this analysis, evaluating a decade of FDA approvals, we found that for the vast majority of SAS approvals, it was not only feasible to conduct RCTs but they could also likely be completed within a reasonable duration. Surprisingly, 5 of 31 drugs were approved despite an inferior efficacy outcome compared with the existing standard of care while also lacking any apparent practical advantages of using those drugs, raising questions about the rationale for such approvals. The impetus behind this exercise is to direct attention to strategies that could strengthen the current drug-approval system. The results reported here are intended to be thought provoking rather than definitive, given the multiple reasonable assumptions required for the exercise.

Common malignancies accounted for most of the approvals based on SAS, which is directly against the spirit of FDA guidance of AA in SAS. Only a few SAS studies evaluated truly rare cancers. For example, lung cancer was projected to result in 135 720 deaths in 2020 in the United States, yet it was the most frequent tumor type to use SAS (11). Evaluation of targeted treatment for a mutational subtype of NSCLC accounted for most SAS approvals in lung cancer and, hence, was deemed as “rare.” However, the mutational subtypes of *EGFR, ALK* rearrangement, and ROS-1 constitute approximately 17%, 7%, and 2% of all NSCLC, respectively (12). This translates to 19 611, 8075, and 1154 NSCLC annual deaths with these subtypes, respectively, in 2020 alone, resulting in sufficient patients to conduct proper RCTs.

We found that 38.7% of SAS approvals did not report on DOR resulting in drugs being approved based on ORR alone. ORR simply measures biologic activity of the drug and not necessarily a meaningful benefit to patients. Responses could have lasted for a week, a month, or a year, which robs patients of arguably the most crucial information required for informed shared decision making. Of further concern, previous studies have reported that ORR in nonrandomized studies is, on average, 2.5-fold higher compared with those seen in RCTs for the same study drug (6,13). Immunotherapy consisted of close to half of all approvals based on ORR, despite a particularly poor correlation of ORR to early PFS or OS for these agents (14,15). The absence of DOR in a SAS with ORR as primary endpoint appears difficult to justify.

In a highly competitive industry, the quickest route to provisional drug approval is obviously the most desirable, even if this bypasses the historical safeguards established by regulators. From patient and societal perspectives, there are serious trade-offs with this approach with potential for harm to both the rigor of the science and the patients. Lost opportunity for proper evaluation of drugs after AA cannot be overstated. Ribeiro et al. (16) compared FDA AA with regular approvals between 2006 and 2018 and found that the criteria for granting AA were not clear, with AA allowing for more uncertainty in results; AA was much more likely to be based on a SAS. No wonder many drugs that receive AA do not complete the requirement of a definitive RCT even years after receiving such approvals. An evaluation of 25 years of FDA approvals showed that as many as 40% of drugs that received AA had not completed confirmatory trials at the time of their analysis (17). In April 2021, the FDA Oncologic Drug Advisory Committee met and reviewed 6 drugs approved by AA for which confirmatory trials failed to demonstrate expected clinical benefit to date. These include atezolizumab with *nab*-paclitaxel in metastatic triple-negative breast cancer, nivolumab and pembrolizumab for hepatocellular carcinoma, and pembrolizumab and atezolizumab in cisplatin ineligible metastatic urothelial carcinoma (18-22). However, without supporting confirmatory trials, the advisory committee voted to uphold AA for 5 out of 6 reviews stating “unmet need” as the common reason for such decision. Final recommendation from FDA on this topic awaits.

Even absence of an effective standard of care may not be sufficient justification to use SAS for drug approvals, as a RCT may be completed with the control arm of best supportive care or placebo. Notably, an alternate treatment that could be used as a control arm was found for all but 3 approvals. Although the FDA rarely specifies acceptable comparator(s), having a legitimate comparator is exceptionally important to engender trust in the approval process and understand the additional value of a new treatment (23).

We acknowledge that SAS are typically easier to accrue to, because everybody receives the experimental treatment. Such studies are usually conducted at centers with a proven track record for accruing large numbers. Follow-up time in SAS is typically shorter than RCTs, which is one of the reasons RCTs cost substantially more than SAS—another reason behind the industry’s preference of SAS over RCTs. However, SAS are also limited by the use of surrogate endpoints, predominantly assessing ORR with or without DOR, and rarely evaluate OS or quality-of-life endpoints (24). Nonetheless, there are occasions when SAS is the study of choice including when diseases or mutational aberrations are very rare, making patient accrual for RCTs difficult if not infeasible (6). SAS can also be considered when RCT accrual is felt to not be possible. This may occur when there is no previous standard of care and placebo would be the control arm, such as with vismodegib for advanced or metastatic basal cell carcinoma, which received FDA approval in 2012.

Generally, it is assumed that the sample size needed for SAS compared with RCTs is substantially smaller. We estimated the eligible population based on total annual deaths in the United States, however, this would be higher for less lethal cancers and in earlier lines of therapy. Additionally, the SEER database provides a close estimate of cancer statistics, and like any registry, underreporting is a caveat. Although difficulties exist with patient accrual to RCTs, here, duration to complete RCT was calculated based on the conservative accrual rate of 5% of all eligible US patients (25). Recent data suggest that enrollment into clinical trials is improving with estimated accrual of about 16% in academic centers and 7% in the community (7,26). Additionally, RCTs may have international enrollment further increasing the eligible population.

Other limitations of this study lie in the fact that we had to make multiple assumptions to perform the sample size calculations, estimates of accrual rate, and follow-up duration, although most contemporary pivotal RCTs match our assumptions. This study was a simulation exercise of hypothetical RCTs, hence, a thought experiment. The numbers used in our estimates are best approximates based on available knowledge. Our results are meant to be taken seriously, rather than literally—it is more about the message.

All hypothetical RCTs were designed as superiority trials; if a noninferiority trial had to be designed, then hypothetical sample sizes would have also differed (27). Additionally, if a treatment is felt to be futile, at interim analysis, then a trial could be stopped early, requiring a smaller sample size than originally proposed (28). Follow-up duration for the time-to-event endpoints for hypothetical RCTs was set as twice the median life expectancy for the disease condition. This was an arbitrary duration but was considered a conservative assumption. Additionally, our conclusion about timeline for conduct of RCTs is mainly derived from accrual rate alone, as it is hard to quantify delays because of practical issues in the conduct of RCTs.

Here, we present a feasibility assessment conducting hypothetical RCTs for all FDA approvals based on SAS in the last decade, the first empiric assessment of its kind to our knowledge. The results in this study represent a thought experiment which demonstrates that for a large majority of approvals, based on SAS, RCTs may have been feasible within a reasonable time frame. Instances of potential questionable use of the AA pathway were also observed including the use of SAS for relatively common malignancies and approval of drugs despite outcomes inferior to existing standards of care. Early access to drugs for patients is important and can generally be achieved without adversely affecting the population or reducing the rigor of the science conducted. Based on these results, we feel that stronger and clearer criteria for drug approval based on SAS should be mandated by regulatory agencies including the FDA. Additionally, the FDA should reevaluate criteria for AA because this path to approval may be used inappropriately, to the detriment of patients and the system.

## Funding

This work received no external funding.

## Notes

**Role of the funder:** Not applicable.

**Disclosures:** Authors do not have relevant conflicts of interest.

**Author contributions:** RR: Data curation, Formal Analysis, Investigation, Methodology, Project administration, Software, Validation, Visualization, Writing—original draft. PC: Investigation, Methodology, Project administration, Supervision, Validation, Visualization, Writing—review & editing. SN: Conceptualization, Data curation, Formal Analysis, Investigation, Methodology, Project administration, Resources, Software, Supervision, Validation, Visualization, Writing—original draft, Writing—review & editing.

## Data Availability

The data underlying this article will be shared on reasonable request to the corresponding author
